# New Antibacterial Phenone Derivatives Asperphenone A–C from Mangrove-Derived Fungus *Aspergillus* sp. YHZ-1

**DOI:** 10.3390/md16020045

**Published:** 2018-01-30

**Authors:** Zhi-Kai Guo, Yi-Qin Zhou, Hao Han, Wen Wang, Lang Xiang, Xin-Zhao Deng, Hui-Ming Ge, Rui-Hua Jiao

**Affiliations:** 1 State Key Laboratory of Pharmaceutical Biotechnology, Institute of Functional Biomolecules, School of Life Sciences, Nanjing University, Nanjing 210023, China; 15150595842@163.com (Y.-Q.Z.); 15150516577@163.com (H.H.); wangwen7400@163.com (W.W.); xianglang612@yeah.net (L.X.); dengxz2010@163.com (X.-Z.D.); 2 Key Laboratory of Biology and Genetic Resources of Tropical Crops, Ministry of Agriculture, Institute of Tropical Bioscience and Biotechnology, Chinese Academy of Tropical Agricultural Sciences, Haikou 571101, China; guozhikai@itbb.org.cn

**Keywords:** mangrove endophyte, *Aspergillus* sp., marine natural product, asperphenone, antibacterial activity

## Abstract

Marine fungi are a promising source of novel bioactive natural products with diverse structure. In our search for new bioactive natural products from marine fungi, three new phenone derivatives, asperphenone A–C (**1**–**3**), have been isolated from the ethyl acetate extract of the fermentation broth of the mangrove-derived fungus, *Aspergillus* sp. YHZ-1. The chemical structures of these natural products were elucidated on the basis of mass spectrometry, one- and two-dimensional NMR spectroscopic analysis and asperphenone A and B were confirmed by single-crystal X-ray crystallography. Compounds **1** and **2** exhibited weak antibacterial activity against four Gram-positive bacteria, *Staphylococcus aureus* CMCC(B) 26003, *Streptococcus pyogenes* ATCC19615, *Bacillus subtilis* CICC 10283 and *Micrococcus luteus*, with the MIC values higher than 32.0 µM.

## 1. Introduction

Marine filamentous fungi are a rich source of antimicrobial compounds, anti-inflammatory, anticancer and antiviral agents [1,2,3,4,5]. Among all the marine-sourced fungi, the species in the genus *Aspergillus* (Trichocomaceae) are an important source for novel pharmacological metabolites such as polyketides, alkaloids and terpenoids [6,7,8,9]. The mangrove plants inhabit the intertidal zones in the tropics and subtropics, providing a very unique habitat for animals and microbes. Thus, the mangrove-associated fungi, with the majority coming from endophytic species, have attracted much attention to discover structurally diverse and bioactive secondary metabolites. In the past several years, our research group has explored many novel secondary metabolites from mangrove-derived endophytic fungi, such as sesquiterpenoids diaporols A-I, diaporine with regulation activity of macrophage differentiation and other polyketides [10,11,12]. As part of our ongoing search for novel biologically active natural products from microbe from special environment [13,14], we chemically investigated an endophytic fungus, *Aspergillus* sp. YHZ-1, from mangrove plant from Hainan Island, China. Fractionation of the ethyl acetate extract of its liquid fermentation broth led to the discovery of three new phenone derivatives, which we have named asperphenone A–C (**1**–**3**). Compounds **1** and **2** were tested and displayed weak antibacterial activity against four Gram-positive bacteria. Herein, we report the isolation and structure elucidation of these new compounds and their antibacterial activity.

## 2. Results

**Figure 1:**
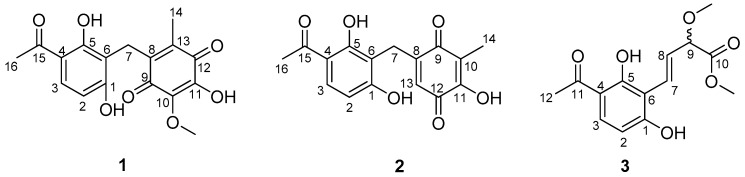
The structures of asperphenone A–C (**1**–**3**) isolated from *Aspergillus* sp. YHZ-1.

A large-scale fermentation broth (50 L) of *Aspergillus* sp. YHZ-1 was collected and extracted with ethyl acetate three times to produce a crude extract. The subsequent fractionation by repeated column chromatography over silica gel, octadecylsilyl silica gel (ODS), Sephadex LH-20 and semi-preparative reversed-phase high performance liquid chromatography (HPLC) yielded three new phenone derivatives, asperphenone A–C (**1**–**3**) (Figure 1).

**Figure 2:**
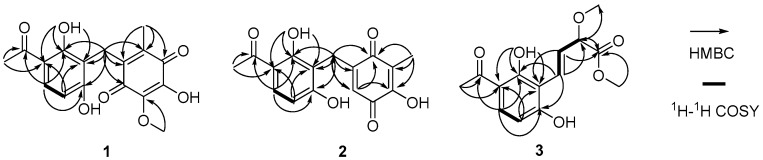
The key 2D NMR correlations of asperphenone A–C (**1**–**3**).

**Figure 3:**
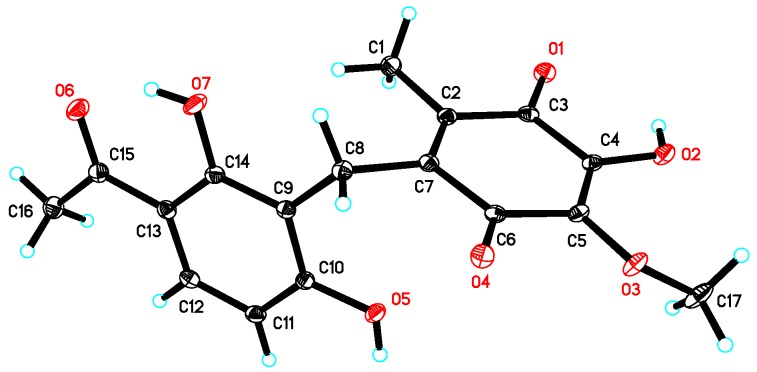
X-ray crystal structure of **1**.

Asperphenone A (**1**) was isolated as a reddish-brown needle crystal. The molecular formula of **1** was determined to be C_17_H_16_O_7_ on the basis of the high-resolution (HR) ESI-MS (*m*/*z* 355.2716 [M + Na]^+^, calcd. 355.2706) along with the ^1^H and ^13^C NMR data (Table 1), indicating ten degrees of unsaturation. The ^1^H, ^13^C and HSQC NMR spectra (in supplementary materials) in DMSO-*d*_6_ revealed signals of two methyls (*δ*_C_/*δ*_H_ 11.6/1.73, C-14/H_3_-14; *δ*_C_/*δ*_H_ 26.6/2.52, C-16/H_3_-16), one oxygenated methyl (*δ*_C_/*δ*_H_ 60.3/3.78, 10-OMe), one methylene (*δ*_C_/*δ*_H_ 19.4/3.70, C-7/H_2_-7), one ketone carbon (*δ*_C_ 203.8, C-15), two *α*,*β*-unsaturated carbonyl carbons (*δ*_C_ 182.7, C-9; *δ*_C_ 185.1, C-12), three phenolic hydroxyl protons (*δ*_H_ 10.25, 1-OH; *δ*_H_ 13.21, 5-OH; *δ*_H_ 10.85, 11-OH) and ten aromatic/olefinic carbons, whose chemical shift values indicated the presence of one 1,2,3,4-tetrasubstituted benzene ring (*δ*_C_ 162.7, C-1; *δ*_C_/*δ*_H_ 107.8/6.42 (d, *J* = 8.8 Hz), C-2/H-2; *δ*_C_/*δ*_H_ 131.8/7.67 (d, *J* = 8.8 Hz), C-3/H-3; *δ*_C_ 112.8, C-4; *δ*_C_ 162.4, C-5; *δ*_C_ 112.0, C-6) and four quaternary carbons (*δ*_C_ 136.0, C-8; *δ*_C_ 138.8, C-10; *δ*_C_ 143.3, C-11; *δ*_C_ 144.5, C-13). The tetrasubstituted benzene ring was determined as a 1,3-dihydroxy-4-acetophenone moiety, which was deduced by analysis of the ^1^H-^1^H COSY correlation from H-2 to H-3 and the HMBC correlations from H-2 to C-4 and C-6, from H-3 to C-1, C-5 and C-15, from H_3_-16 to C-4 and C-15 and from 5-OH to C-4, C-5 and C-6 (Figure 2). HMBC correlations from H_2_-7 to C-1, C-5 and C-6 permitted the linkage of C-7 to C-6 of the acetophenone moiety. The presence of a *p*-benzoquinone moiety could be deduced from the chemical shifts of C8-C13 in **1**, which was partially confirmed by the observation of HMBC correlations from H_3_-14 to C-8, C-12 and C-13 and from H_2_-7 to C-8, C-9 and C13. Meanwhile, the connectivity of the *p*-benzoquinone and acetophenone moieties via the methylene C-7 was established by the relevant HMBC correlations. However, the positions of the hydroxyl group and methoxyl group on the *p*-benzoquinone moiety cannot be determined as their 2D correlations cannot be observed. Finally, a high-quality single crystal of **1** was obtained and the full structure of **1** was determined by the X-ray crystallographic analysis in a Cu K*α* radiation in low temperature (Figure 3), which also confirmed the proposed structure of **1**.

**Figure 4:**
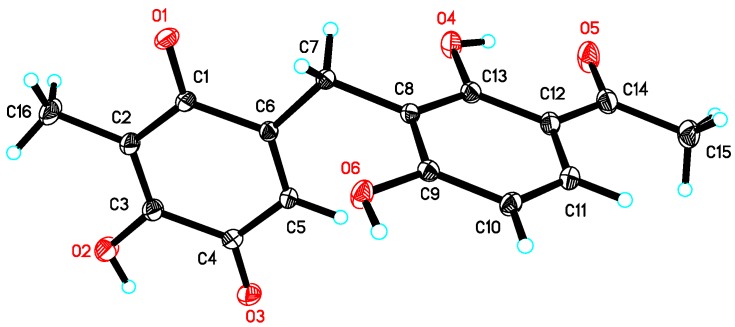
X-ray crystal structure of **2**.

Asperphenone B (**2**) was obtained as a yellow needle crystal, with the molecular formula C_16_H_14_O_6_ (ten degrees of unsaturation) as derived from the HRESIMS data (*m*/*z* 325. 1326 [M + Na]^+^, calcd. 325.0683). The ^13^C NMR spectrum showed sixteen carbon signals for two methyls (C-14 and C-16), one methylene (C-7), one ketone carbon (C-15), two *α*,*β*-unsaturated carbonyl carbons (C-9 and C-12) and ten aromatic/olefinic carbons. Comparison of the MS, ^1^H and ^13^C NMR data of **2** with those of **1** revealed the presence of the same 1,3-dihydroxy-4-acetophenone moiety as **1** and a different substituted *p*-benzoquinone moiety. Further HMBC correlations (Figure 2) from H_3_-14 (*δ*_H_ 1.82) to C-9 (*δ*_C_ 187.2), C-10 (*δ*_C_ 116.9) and C-11(*δ*_C_ 153.8), from H-13 (*δ*_H_ 5.76) to C-7 (*δ*_C_ 22.0), C-8 (*δ*_C_ 148.5), C-9 and C-11 and from H_2_-7 (*δ*_H_ 3.61) to C-8, C-9 and C-13 (*δ*_C_ 127.1) along with the unsaturation requirement of **2** confirmed the substituted pattern of the *p*-benzoquinone moiety. Finally, the connection between the two moieties mentioned above was through C-7 by interpretation of the relevant HMBC correlations from H_2_-7. Detailed NMR analysis allowed the assignment of **2** as shown in Figure 1, which was also confirmed by a low-temperature single-crystal X-ray diffraction experiment (Figure 4).

Asperphenone C (**3**) was isolated as a colorless needle crystal that analyzed for the molecular formula C_14_H_16_O_6_ (seven degrees of unsaturation) by HRESIMS (*m*/*z* 303. 1215 [M + Na]^+^, calcd. 303.0839) in combination with ^13^C NMR data (Table 1). The ^13^C and DEPT135 NMR spectra of **3** displayed signals for fourteen carbons, including one methyl carbon (*δ*_C_ 26.3, C-12), two oxygenated methyl carbons (*δ*_C_ 57.0, 9-OMe; *δ*_C_ 52.0, 10-OMe), one oxygenated methine (*δ*_C_ 83.3, C-9), one ketone carbon (*δ*_C_ 204.3, C-11), one carboxylic carbon (*δ*_C_ 171.7, C-10) and eight aromatic/olefinic carbons. In this molecule, the same moiety, 1,3-dihydroxy-4-acetophenone as found in **1**, could be deduced through analysis of the ^1^H-^1^H COSY correlation from H-2 (*δ*_H_ 6.55, d, *J* = 8.6 Hz) to H-3 (*δ*_H_ 7.71, d, *J* = 8.6 Hz) and HMBC correlations from H-2 to C-4 (*δ*_C_ 113.8) and C-6 (*δ*_C_ 111.4), from H-3 to C-1 (*δ*_C_ 163.3), C-5 (*δ*_C_ 164.4) and C-11, from 5-OH (*δ*_H_ 13.73) to C-4, C-5 and C-6 and from H_3_-12 (*δ*_H_ 2.56) to C-4 and C-11. ^1^H-^1^H COSY correlations from H-7 (*δ*_H_ 7.07, dd, *J* = 16.2, 0.8 Hz) to H-8 (*δ*_H_ 6.78, dd, *J* = 16.2, 7.0 Hz) and from H-8 to H-9 (*δ*_H_ 4.42, dd, *J* = 7.0, 0.8 Hz) and HMBC cross-peaks from H-8 to the carboxylic carbon C-10 established the fragment C-7-C-8-C-9-C-10, which was attached to the acetophenone moiety through C-6-C-7 linkage on the basis of the HMBC correlations from H-8 to C-6 and from H-7 to C-1 and C-5. ^3^*J*_C-H_ diagnostic correlations from the oxygenated methyl protons at *δ*_H_ 3.37 to C-9 and from the oxygenated methyl protons at *δ*_H_ 3.70 to C-10 demonstrated that the methoxyl groups should be located at C-9 and C-10, respectively. Thus, the structure of compound **3** was elucidated as shown in Figure 1. However, the absolute configuration of C-9 has not been determined.

In the primary screen for antibacterial compounds, compounds **1** and **2** were tested against four Gram-positive bacteria, *Staphylococcus aureus* CMCC(B) 26003, *Streptococcus pyogenes* ATCC19615, *Bacillus subtilis* CICC 10283 and *Micrococcus luteus*. As summarized in Table 2, Both of these two compounds showed weak activity against the tested bacteria, *Staphylococcus aureus* CMCC(B) 26003, *Streptococcus pyogenes* ATCC19615, *Bacillus subtilis* CICC 10283 and *Micrococcus luteus*, with the MIC values higher than 32.0 µM.

## 3. Materials and Methods

### 3.1. General Experimental Procedures

The HR-ESI-MS data were obtained on an Agilent 6210 time of flight LC-MS instrument (Agilent Technologies Inc., Palo Alto, CA, USA). NMR experiments were conducted on a Bruker DPX-400 NMR spectrometer (400 MHz for ^1^H NMR and 100 MHz for ^13^C NMR) or Bruker DRX-600 spectrometer (600 MHz for ^1^H NMR and 150 MHz for ^13^C NMR) (Bruker Corporation, Karlsruhe, Germany). The chemical shifts were given in *δ* (ppm) and referenced to the solvent signal (DMSO-*d*_6_, *δ*_H_ 2.50, *δ*_C_ 39.5; acetone-*d*_6_, *δ*_H_ 2.05, *δ*_C_ 29.8). Column chromatography (CC) was accomplished on silica gel (200–300 mesh, Qingdao Marine Chemical Inc., Qingdao, China), ODS (40–70 µm, Merck Company, Darmstadt, Germany) and Sephadex LH-20 (GE Healthcare Bio-Sciences AB, Uppsala, Sweden). Semi-preparative reverse-phase (RP) HPLC was performed on a Hitachi HPLC system with a L-7110 pump, a L-7420 UV/vis detector and an Hypersil RP-C_18_ column (5 µm, 250 × 10.0 mm, Thermo Fisher Scientific, Waltham, MA, USA). Thin-layer chromatography (TLC) was conducted on silica gel GF254 (10–20 µm, Qingdao Marine Chemical Inc., Qingdao, China). All chemicals used were of HPLC grade or analytical grade.

### 3.2. Strain Isolation and Cultivation

The fungus *Aspergillus* sp. YHZ-1 was isolated by one of the authors (Y.-Q.Z.) from an unidentified mangrove plant from Hainan Island, China, in October 2015. The isolate was identified as *Aspergillus* sp. by its morphological characteristics and the voucher specimen (IFB-YHZ-1) was deposited in the Institute of Functional Biomolecules, State Key Laboratory of Pharmaceutical Biotechnology, Nanjing University. The strain was cultivated on MEA agar plate (consisting of 20 g/L malt extract, 20 g/L sucrose, 1 g/L peptone, 20 g/L agar and deionized water) at 30 °C for 5 days. Then small agar plugs with mycelia were inoculated into five 1 L-Erlenmeyer flasks, each containing 400 mL MEA liquid medium, which were cultivated at 28 °C with 108 rpm/min. After 3 days of fermentation, 10 mL of the seed cultures were inoculated into 200 flasks with 250 mL ME liquid medium and fermented on a rotary shaker with 120 rpm/min at 28 °C for 14 days.

### 3.3. Extraction and Isolation

The entire filtrate of the fermented broth (50 L) was harvested and extracted three times with an equivalent volume of ethyl acetate at room temperature. The organic solvent was evaporated in vacuo to yield 25 g of crude extract. Then the crude extract was fractionated by silica gel (200–300 mesh) CC eluting with a gradient of CH_2_Cl_2_-MeOH mixtures (*v*/*v*, 100:0, 100:1, 100:2, 100:4, 100:8, 100:16, 100:32, 0:100) to give 8 fractions (Fr.1–8). Fr.3 (CH_2_Cl_2_-MeOH, *v*/*v*, 100:2) was subsequently subjected to ODS CC with MeOH-H_2_O (*v*/*v*, 30:70, 40:60, 50:50, 60:40, 70:30, 100:0) to afford 6 sub fractions (Fr.3.1–Fr.3.6). Finally, compounds **1** (12.5 mg), **2** (11.2 mg), **3** (6.3 mg) were purified from Fr.3.4 and Fr.3.5 by semi-preparative reverse-phase HPLC (2 mL/min, detector UV *λ*_max_ 254 nm, CH_3_CN-H_2_O 55:45).

### 3.4. Crystal Data of ***1***

The single crystal X-ray diffraction data of compound **1** was collected on a Bruker APEX-II diffractometer at 130 K with Cu K*α* radiation (*λ* = 1.54178 Å). The structure was solved using the program SHELXS-97 and refined by full-matrix least-squares on *F*^2^. Crystal data of compound **1** have been deposited with the Cambridge Crystallographic Data Centre (deposition No. CCDC 1584390), which can be obtained free of charge via www.ccdc.cam.ac.uk/data_request/cif.

Crystal data for **1**: molecular formula C_17_H_16_O_7_, *M_r_* = 332.30, monoclinic crystals, *a* = 13.2620 (3) Å, *b* = 16.2202 (4) Å, *c* = 7.0447 (2) Å, *α* = 90.00°, *β* = 97.7510° (10), *γ* = 90.00°, *Z* = 4, *μ* = 0.977 mm^−1^, *F* (000) = 696 and *T* = 130 K; Crystal dimensions: 0.15 × 0.1 × 0.05 mm^3^, Volume = 1501.56 (7) Å^3^, 10,794 reflections measured, 2783 independent reflections (*R_int_* = 0.0472), the final *R* indices [*I* > 2*σ*(*I*)] *R*_1_ = 0.0469, *wR*_2_ *=* 0.1286, *R* indices (all data) *R*_1_ = 0.0500, *wR*_2_ *=* 0.1330. The goodness of fit on *F*^2^ was 1.026.

### 3.5. Crystal Data of ***2***

The single crystal data of compound **2** was collected on a Bruker APEX-II diffractometer at 130 K with Cu K*α* radiation (*λ* = 1.54178 Å). The structure was solved using the program SHELXS-97 and refined by full-matrix least-squares on *F*^2^. Crystal data of compound **2** have been deposited with the Cambridge Crystallographic Data Centre (deposition No. CCDC 1584387), which can be obtained free of charge via www.ccdc.cam.ac.uk/data_request/cif.

Crystal data for **2**: molecular formula C_16_H_14_O_6_, *M_r_* = 302.27, monoclinic crystals, *a* = 11.4611 (4) Å, *b* = 4.9487 (2) Å, *c* = 13.6864 (5) Å, *α* = 90.00°, *β* = 111.933° (2), *γ* = 90.00°, *Z* = 2, *μ* = 0.909 mm^−1^, *F* (000) = 316 and *T* = 130 K; Crystal dimensions: 0.22 × 0.2 × 0.15 mm^3^, Volume = 720.07 (5) Å^3^, 4697 reflections measured, 1843 independent reflections (*R_int_* = 0.0350), the final *R* indices [*I* > 2*σ*(*I*)] *R*_1_ = 0.0385, *wR*_2_ *=* 0.1115, *R* indices (all data) *R*_1_ = 0.0389, *wR*_2_ *=* 0.1119. The goodness of fit on *F*^2^ was 1.095.

### 3.6. Antibacterial Activity Assay

The in vitro antibacterial activity of compounds **1** and **2** were evaluated against four bacteria including *Staphylococcus aureus* CMCC(B) 26003, *Streptococcus pyogenes* ATCC19615, *Bacillus subtilis* CICC 10283 and *Micrococcus luteus* in accordance with previously reported methods [14,15]. In the assays, the medium used in the test was Müller-Hinton (MH) broth. All test compounds and the positive control ampicillin were dissolved in dimethyl sulfoxide (DMSO). The minimum inhibitory concentration (MIC) values were determined in the 96-well plates (triplicate) and determined as the lowest sample concentration exhibiting no bacterial growth.

## 4. Conclusions

Three new phenone derivatives, asperphenone A–C (**1**–**3**), were isolated from the ethyl acetate extract of the fermentation broth of the mangrove-derived fungus *Aspergillus* sp. YHZ-1. The chemical structures of these compounds were elucidated on the basis of HR-ESI-MS, 1D and 2D NMR spectroscopic analysis, as well as X-ray crystallographic data. Both of the tested compounds **1** and **2** displayed weak antibacterial activity against four Gram-positive bacteria, *Staphylococcus aureus* CMCC(B) 26003, *Streptococcus pyogenes* ATCC19615, *Bacillus subtilis* CICC 10283 and *Micrococcus luteus*, indicating that the mangrove-associated fungi are still a rich source for discovering diverse new bioactive natural products which could be used as lead compounds in drug development.

## Figures and Tables

**Table 1:** ^1^H and ^13^C NMR data for compounds **1**–**3**.

No.	1 ^a^		2 ^a^		3 ^b^	
	*δ*_H_, Mult. (*J* in Hz)	*δ*_C_, Type	*δ*_H_, Mult. (*J* in Hz)	*δ*_C_, Type	*δ*_H_, Mult. (*J* in Hz)	*δ*_C_, Type
1		162.7, C		162.3, C		163.3, C
1-OH	10.25 ^c^, brs		10.75, brs			
2	6.42, d (8.8)	107.8, CH	6.52, d (8.8)	107.4, CH	6.55, d (8.6)	108.3, CH
3	7.67, d (8.8)	131.8, CH	7.77, d (8.8)	132.1, CH	7.71, d (8.6)	132.8, CH
4		112.8, C		112.5, C		113.8, C
5		162.4, C		162.7, C		164.4, C
5-OH	13.21, s		13.09, s		13.73, s	
6		112.0, C		109.5, C		111.4, C
7	3.70, s	19.4, CH_2_	3.61, d (2.0)	22.0, CH_2_	7.07, dd (16.2, 0.8)	124.1, CH
8		136.0 ^d^, C		148.5, C	6.78, dd (16.2, 7.0)	128.6, CH
9		182.7, C		187.2, C	4.42, dd (7.0, 0.8)	83.3, CH
9-OMe					3.37, s	57.0, CH_3_
10		138.8, C		116.9, C		171.7, C
10-OMe	3.78, s	60.3, CH_3_			3.70, s	52.0, CH_3_
11		143.3, C		153.8, C		204.3, C
11-OH	10.85 ^c^, br s		10.75, brs			
12		185.1, C		183.0, C	2.56, s	26.3, CH_3_
13		144.5 ^d^, C	5.76, t (2.0)	127.1, CH		
14	1.73, s	11.6, CH_3_	1.82, s	8.15, CH_3_		
15		203.8, C		203.4, C		
16	2.52, s	26.6, CH_3_	2.55, s	26.2, CH_3_		

^a^ Acquired at 400 MHz for ^1^H NMR and 100 MHz for ^13^C NMR in DMSO-*d*_6_; ^b^ Acquired at 600 MHz for ^1^H NMR and 150 MHz for ^13^C NMR in acetone-*d*_6_; ^c,d^ Interchangeable signals.

**Table 2:** Antibacterial activities of compounds **1** and **2** (MIC, μM).

Compounds	*S. aureus*	*B. subtilis*	*S. pyogenes*	*M. luteus*
**1**	64.0	64.0	64.0	32.0
**2**	32.0	64.0	32.0	32.0
Ampicillin	4.0	8.0	2.0	1.0

## Supplementary Materials

The 1D and 2D NMR spectra for compounds **1**–**3** are available online at http://www.mdpi.com/1660-3397/16/2/45/s1.
