# Supplementary material for: New Antibacterial Phenone Derivatives Asperphenone A–C from Mangrove-Derived Fungus Aspergillus sp. YHZ-1
Source: Mar Drugs. 2018 Jan 30;16(2):45. doi: 10.3390/md16020045 (PMC5852473; doi:10.3390/md16020045)

**Supporting Information**

**New Antibacterial Phenone Derivatives Asperphenone A-C from Mangrove-Derived Fungus *Aspergillus* sp. YHZ-1**

Zhi-Kai Guo ^2,‡^, Yi-Qin Zhou ^1,‡^, Hao Han ^1^, Wen Wang ^1^, Lang Xiang ^1^, Xin-Zhao Deng ^1^, Hui-Ming Ge ^1^,* and Rui-Hua Jiao ^1^,*

^1^ State Key Laboratory of Pharmaceutical Biotechnology, Institute of Functional Biomolecules, School of Life Sciences, Nanjing University, Nanjing 210023, People’s Republic of China; [15150595842@163.com](mailto:15150595842@163.com) (Y.-Q.Z.); 972944168@qq.com (H.H.); wangwen7400@163.com (W.W.); 971649282@qq.com (L.X.); dengxz2010@163.com (X.-Z. D.)

^2^ Key Laboratory of Biology and Genetic Resources of Tropical Crops, Ministry of Agriculture, Institute of Tropical Bioscience and Biotechnology, Chinese Academy of Tropical Agricultural Sciences, Haikou 571101, People’s Republic of China; guozhikai@itbb.org.cn (Z.-K. G.)

***** Correspondence: rhjiao@nju.edu.cn (R.-H.J.); hmge@nju.edu.cn (H.-M.G.)

‡ These authors contributed equally to this work.

Figure S1. ^1^H NMR (400 MHz, DMSO-*d*_6_) spectrum of compound **1**

Figure S2. ^13^C NMR (100 MHz, DMSO-*d*_6_) spectrum of compound **1**

Figure S3. HSQC spectrum of compound **1** in DMSO-*d*_6_

Figure S4. ^1^H-^1^H COSY spectrum of compound **1** in DMSO-*d*_6_

Figure S5. HMBC spectrum of compound **1** in DMSO-*d*_6_

Figure S6. NOESY spectrum of compound **1** in DMSO-*d*_6_

Figure S7. ^1^H NMR (400 MHz, DMSO-*d*_6_) spectrum of compound **2**

Figure S8. ^13^C NMR (100 MHz, DMSO-*d*_6_) spectrum of compound **2**

Figure S9. HSQC spectrum of compound **2** in DMSO-*d*_6_

Figure S10. ^1^H-^1^H COSY spectrum of compound **2** in DMSO-*d*_6_

Figure S11. HMBC spectrum of compound **2** in DMSO-*d*_6_

Figure S12. NOESY spectrum of compound **2** in DMSO-*d*_6_

Figure S13. ^1^H NMR (600 MHz, acetone-*d*_6_) spectrum of compound **3**

Figure S14. ^13^C NMR (150 MHz, acetone-*d*_6_) spectrum of compound **3**

Figure S15. DEPT135 spectrum of compound **3** in acetone-*d*_6_

Figure S16. HSQC spectrum of compound **3** in acetone-*d*_6_

Figure S17. ^1^H-^1^H COSY spectrum of compound **3** in acetone-*d*_6_

Figure S18. HMBC spectrum of compound **3** in acetone-*d*_6_

Figure S1. ^1^H NMR (400 MHz, DMSO-*d*_6_) spectrum of compound **1**


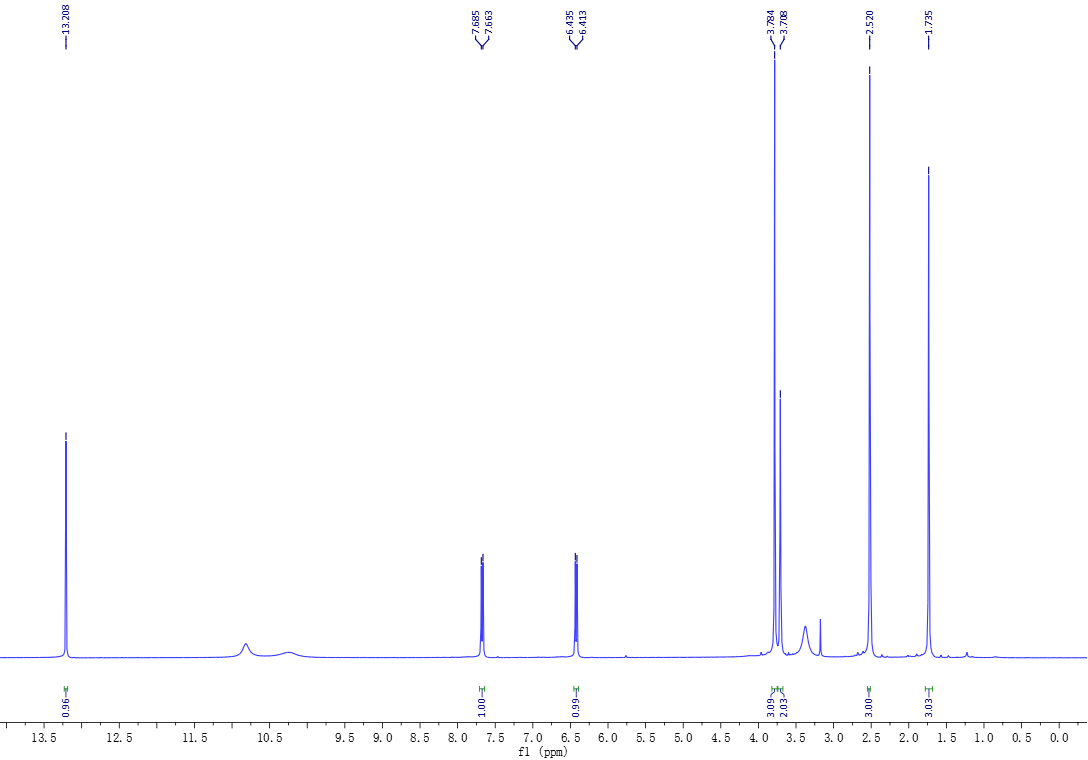


Figure S2. ^13^C NMR (100 MHz, DMSO-*d*_6_) spectrum of compound **1**


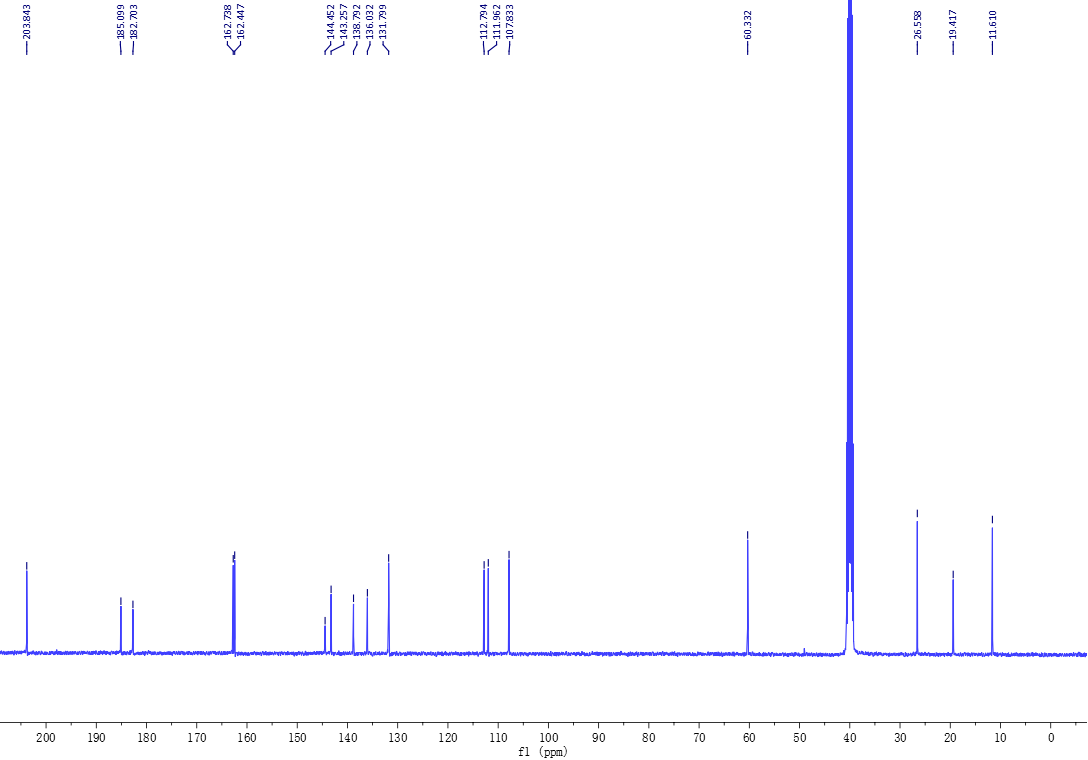


Figure S3. HSQC spectrum of compound **1** in DMSO-*d*_6_


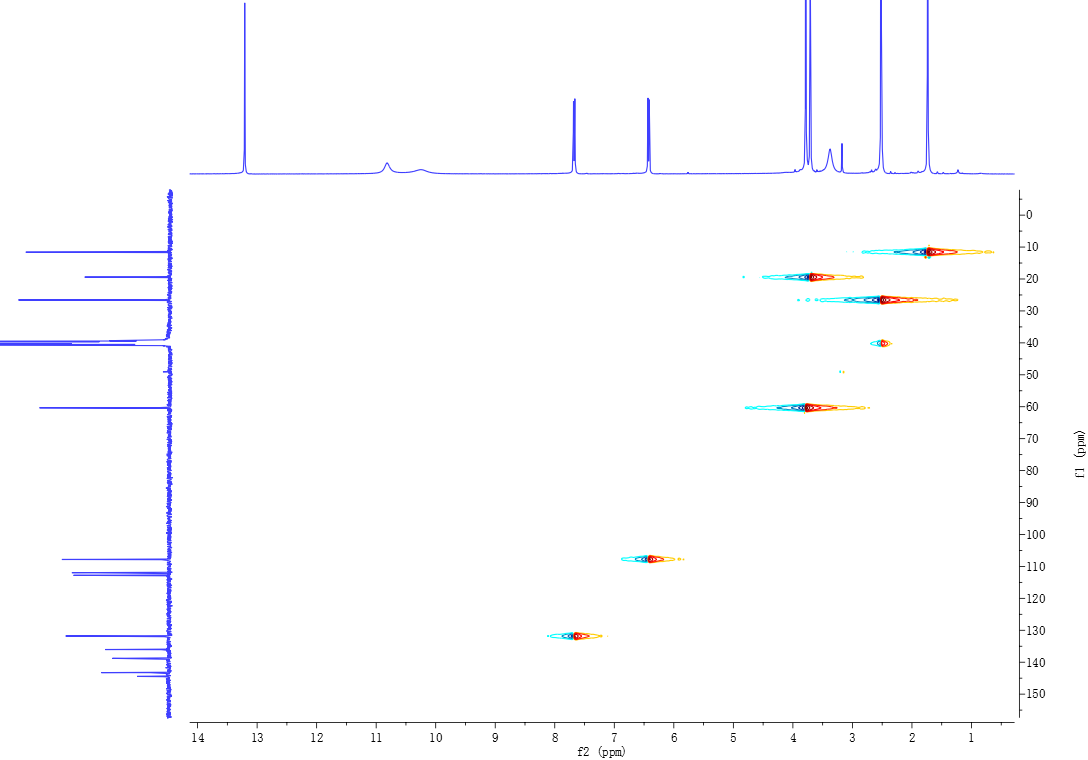


Figure S4. ^1^H-^1^H COSY spectrum of compound **1** in DMSO-*d*_6_


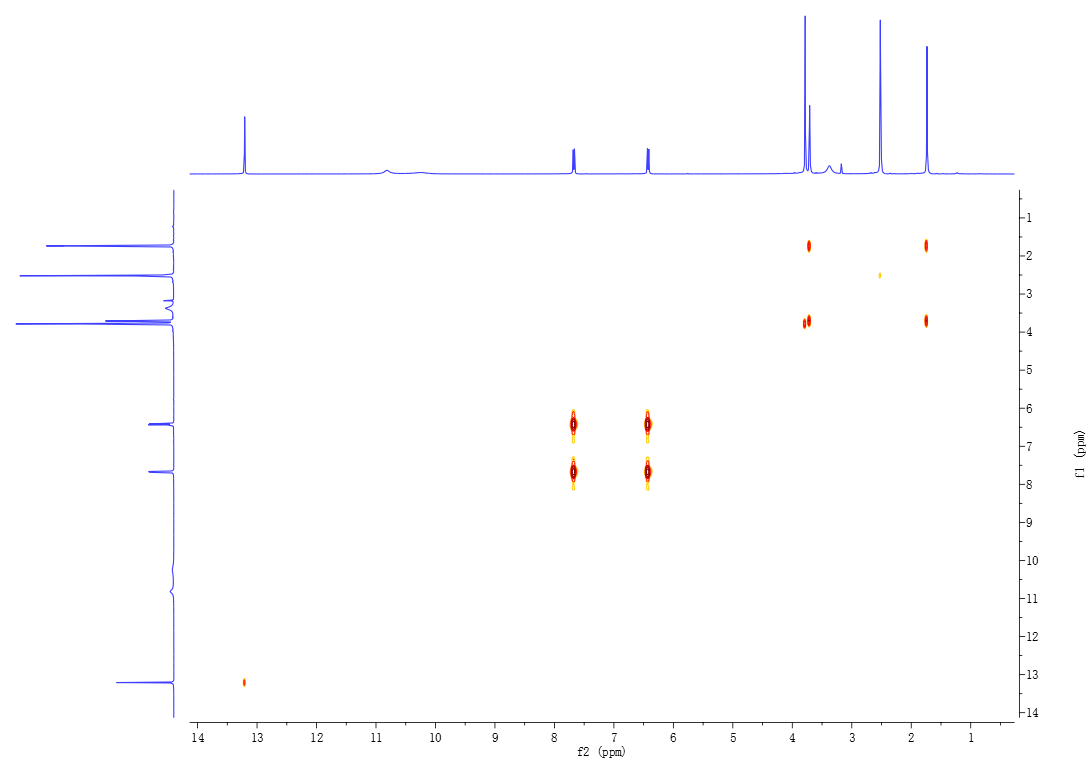


Figure S5. HMBC spectrum of compound **1** in DMSO-*d*_6_


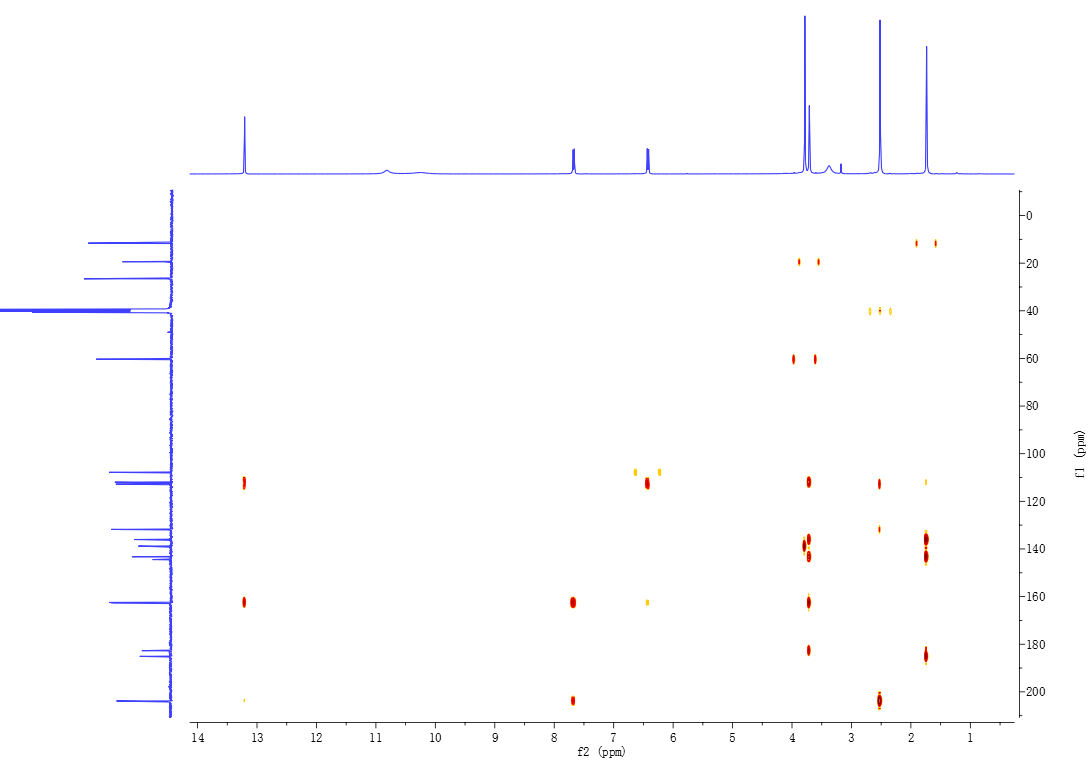


Figure S6. NOESY spectrum of compound **1** in DMSO-*d*_6_


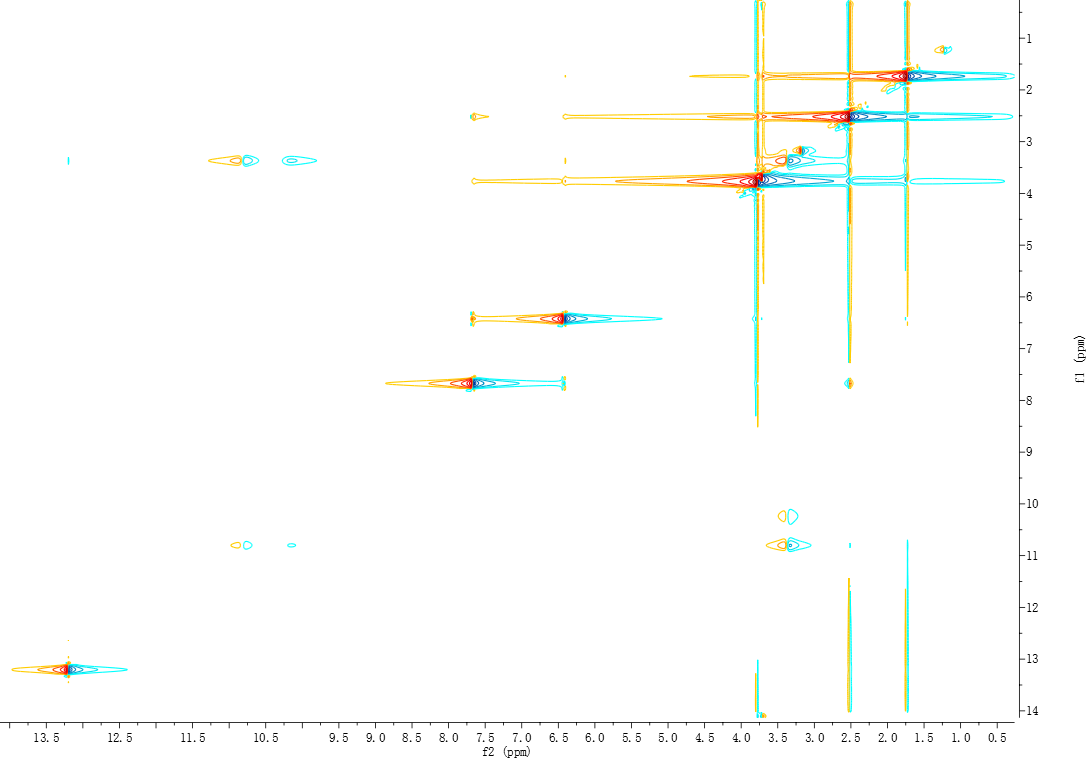


Figure S7. ^1^H NMR (400 MHz, DMSO-*d*_6_) spectrum of compound **2**


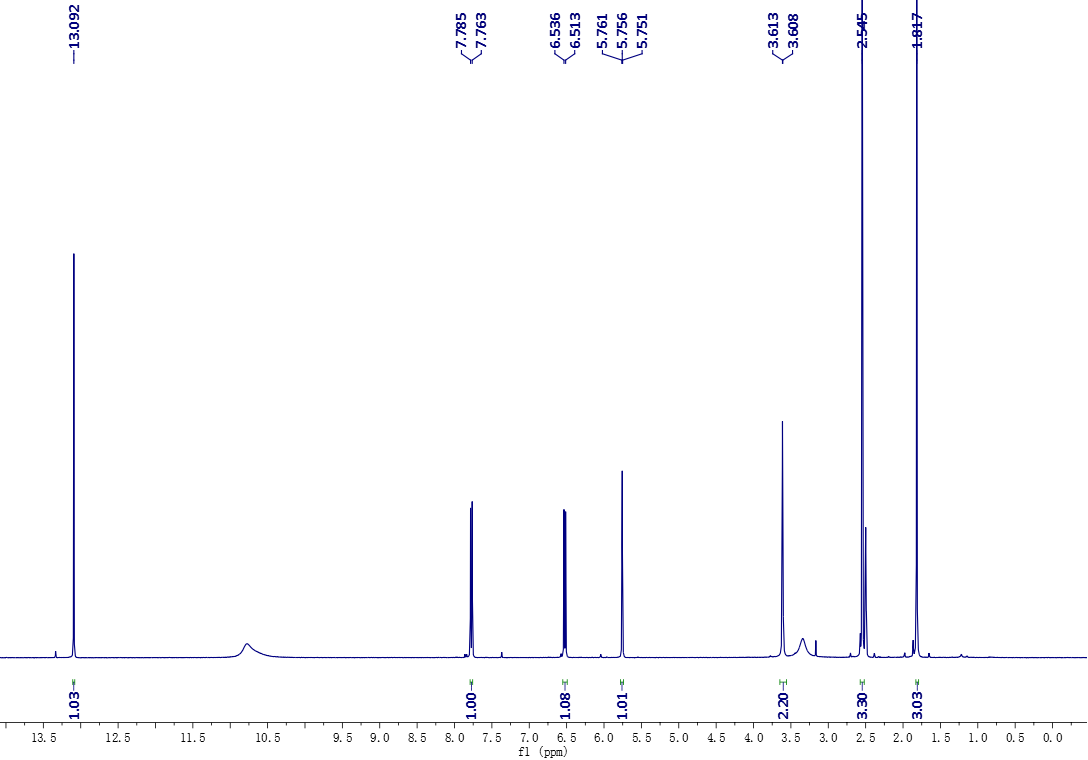


Figure S8. ^13^C NMR (100 MHz, DMSO-*d*_6_) spectrum of compound **2**


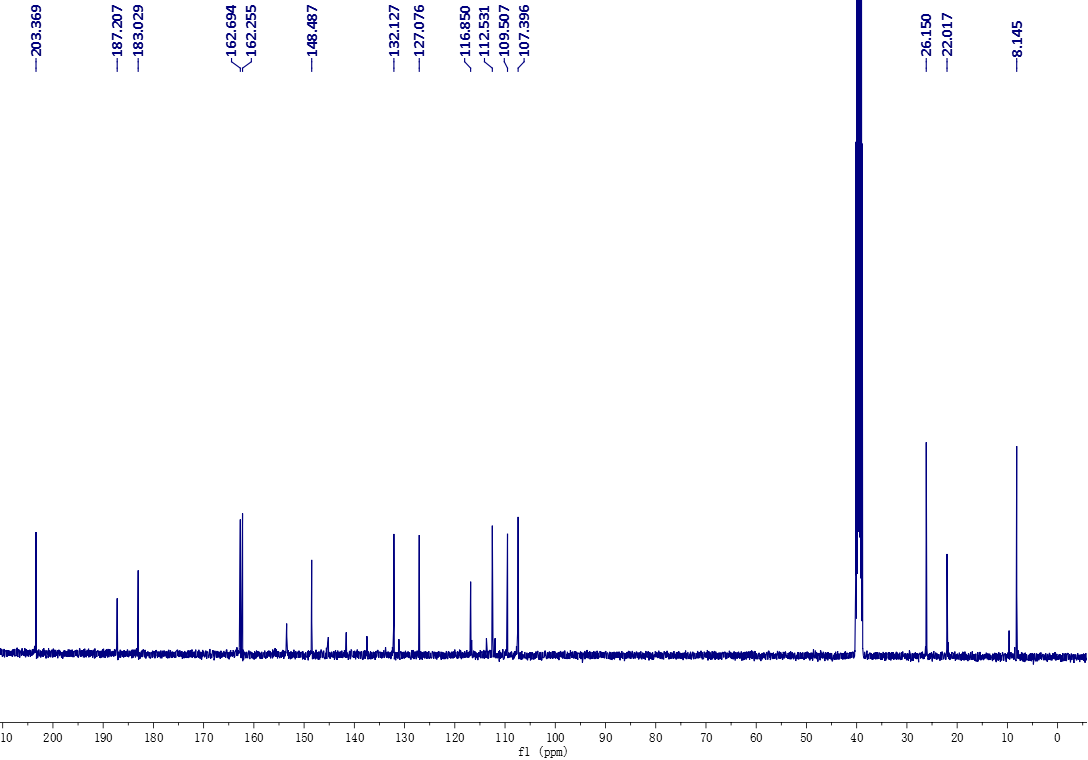


Figure S9. HSQC spectrum of compound **2** in DMSO-*d*_6_


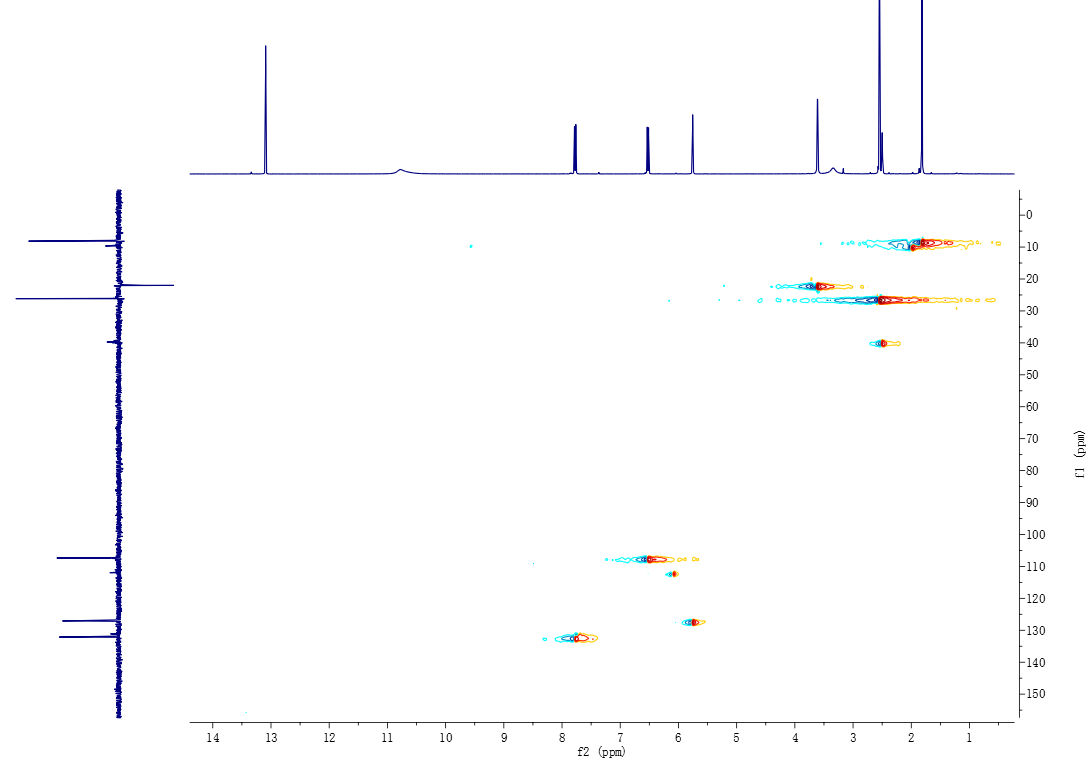


Figure S10. ^1^H-^1^H COSY spectrum of compound **2** in DMSO-*d*_6_


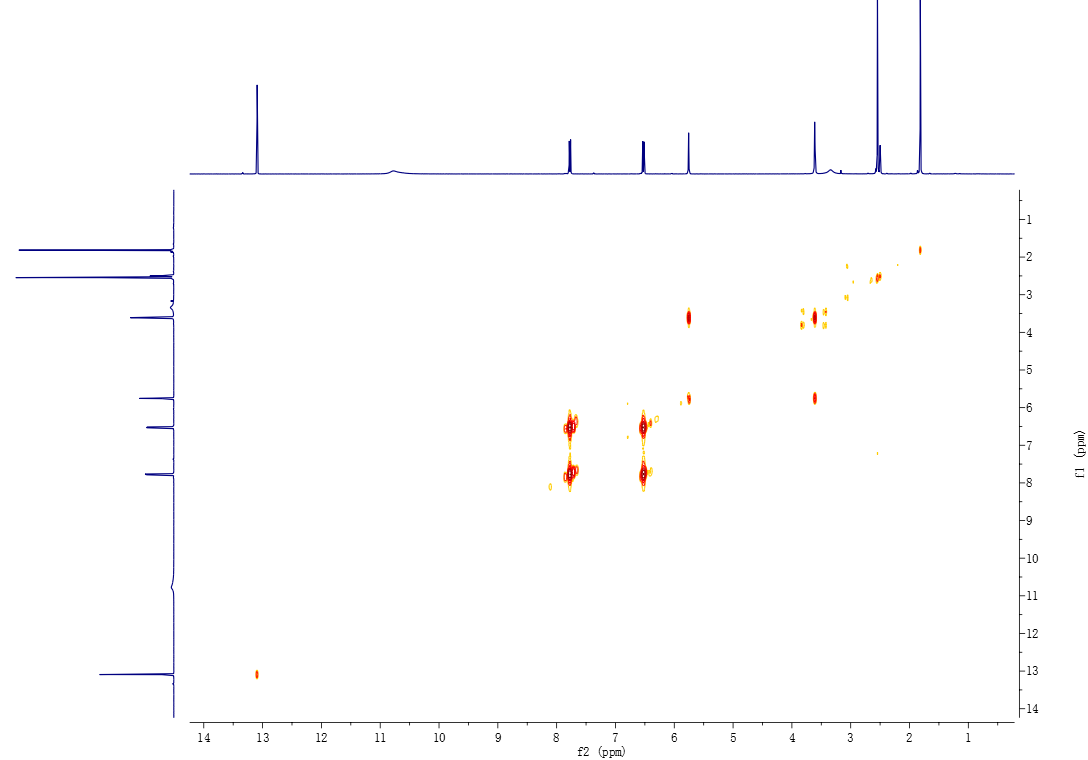


Figure S11. HMBC spectrum of compound **2** in DMSO-*d*_6_


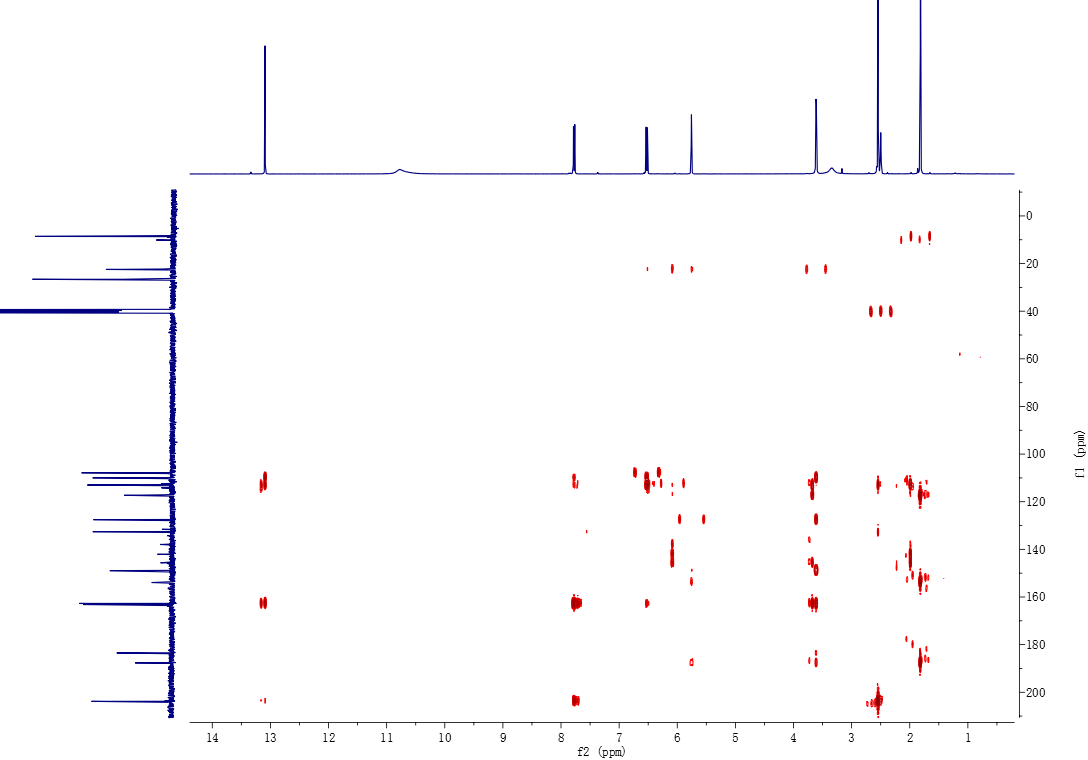


Figure S12. NOESY spectrum of compound **2** in DMSO-*d*_6_


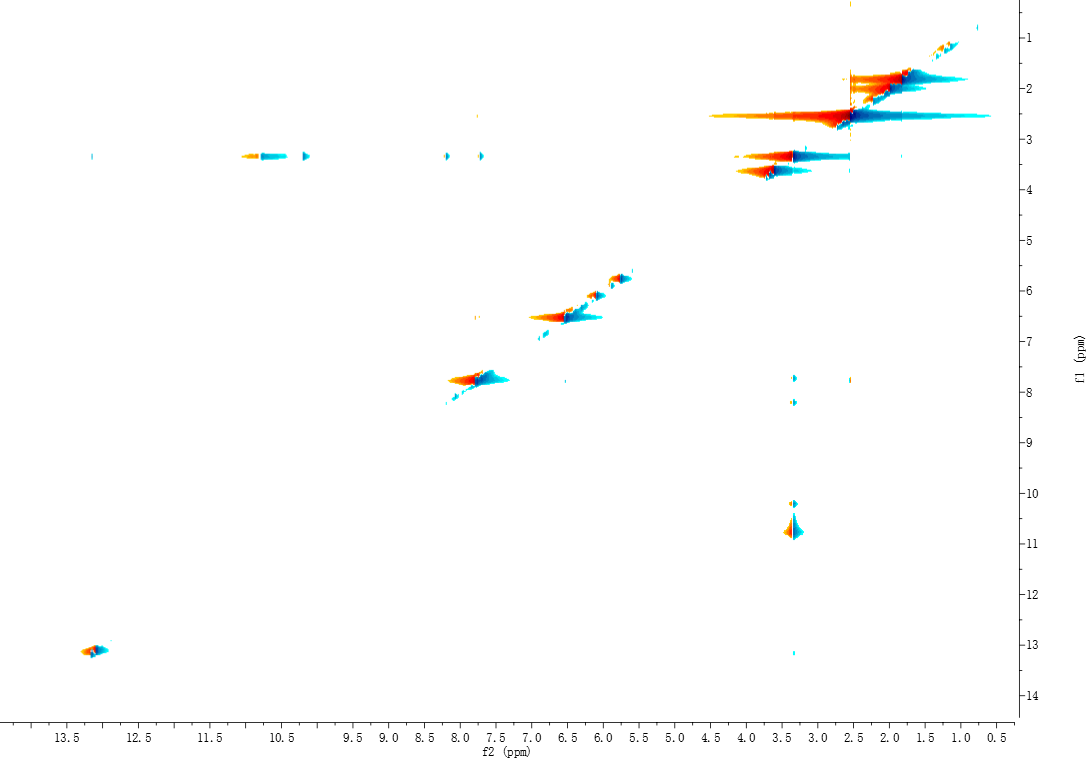


Figure S13. ^1^H NMR (600 MHz, acetone-*d*_6_) spectrum of compound **3**


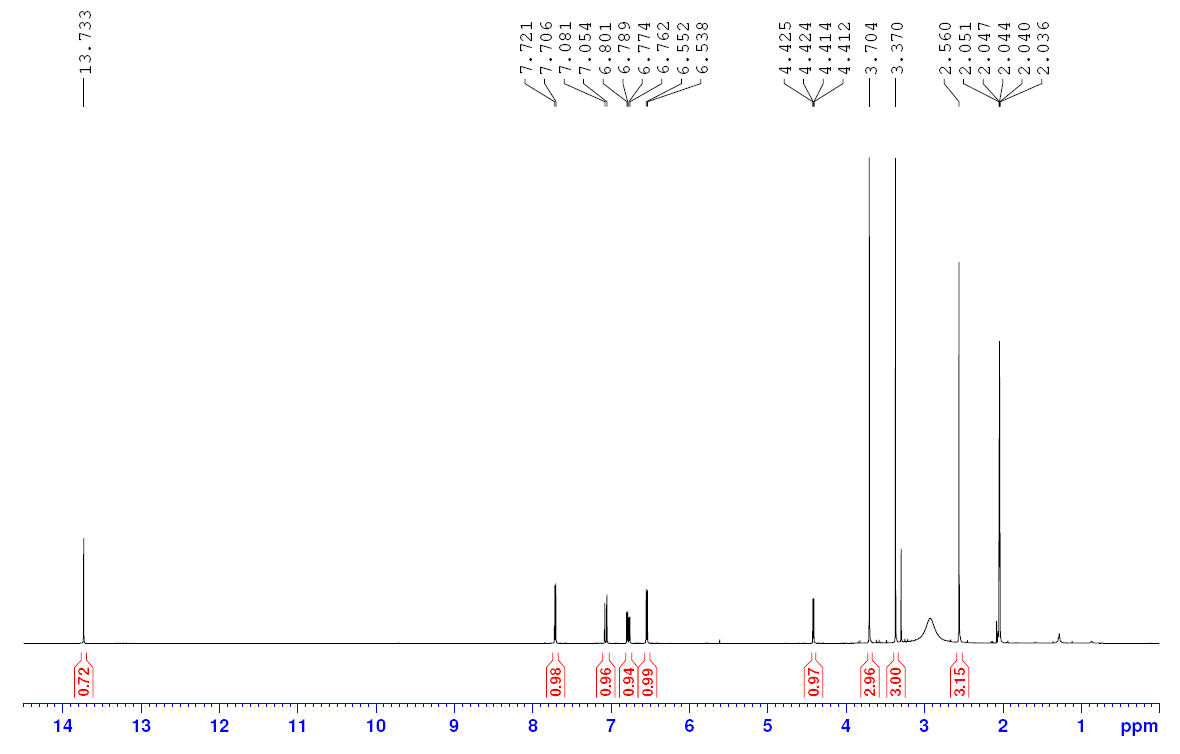


Figure S14. ^13^C NMR (150 MHz, acetone-*d*_6_) spectrum of compound **3**


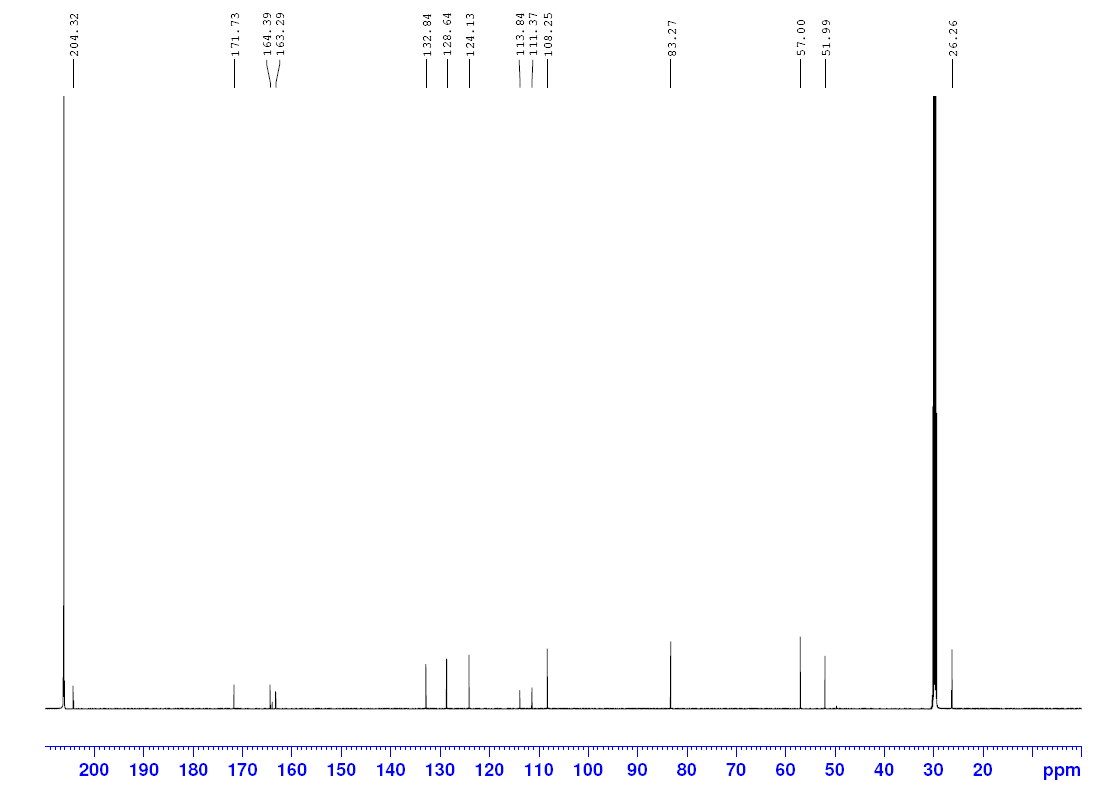


Figure S15. DEPT135 spectrum of compound **3** in acetone-*d*_6_


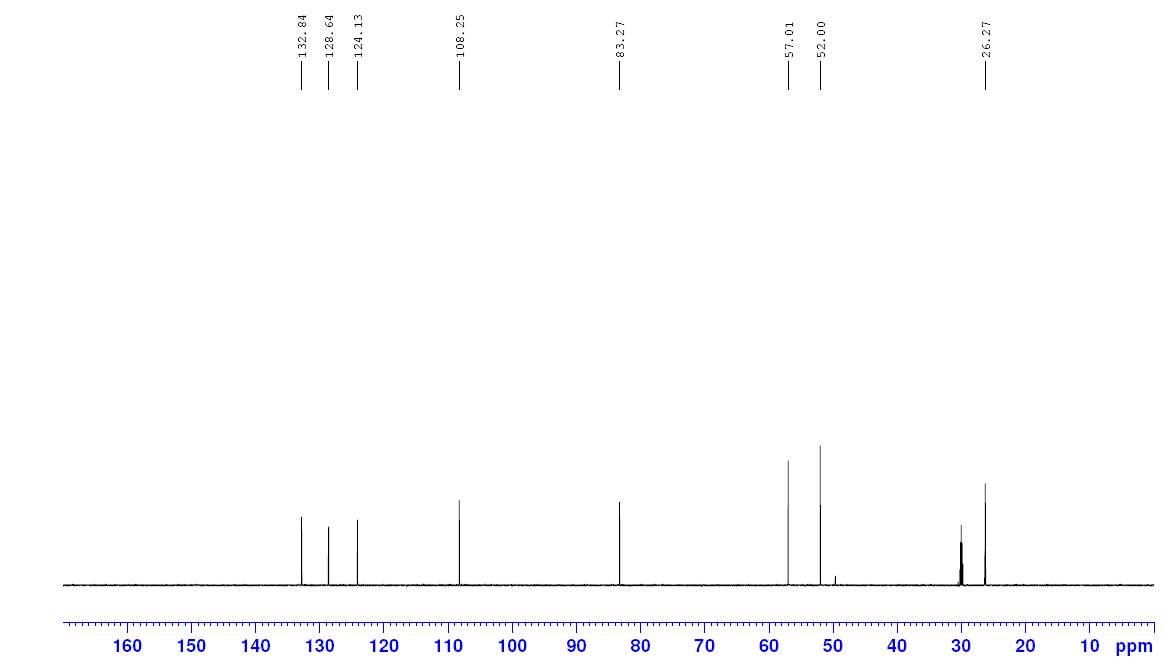


Figure S16. HSQC spectrum of compound **3** in acetone-*d*_6_


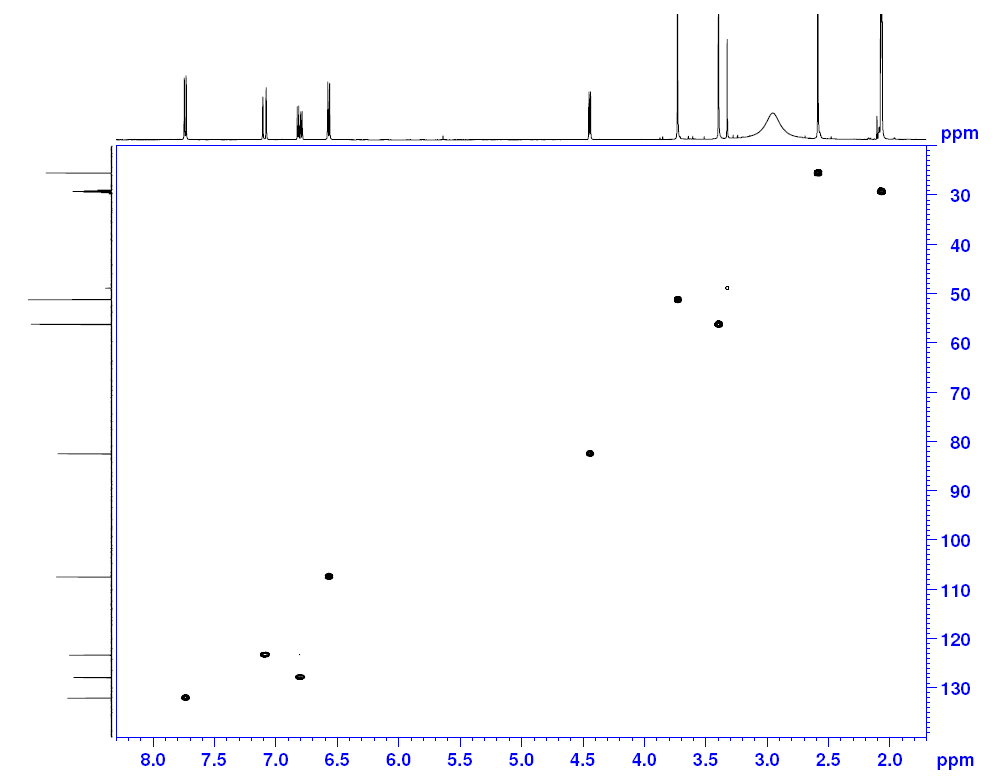


Figure S17. ^1^H-^1^H COSY spectrum of compound **3** in acetone-*d*_6_


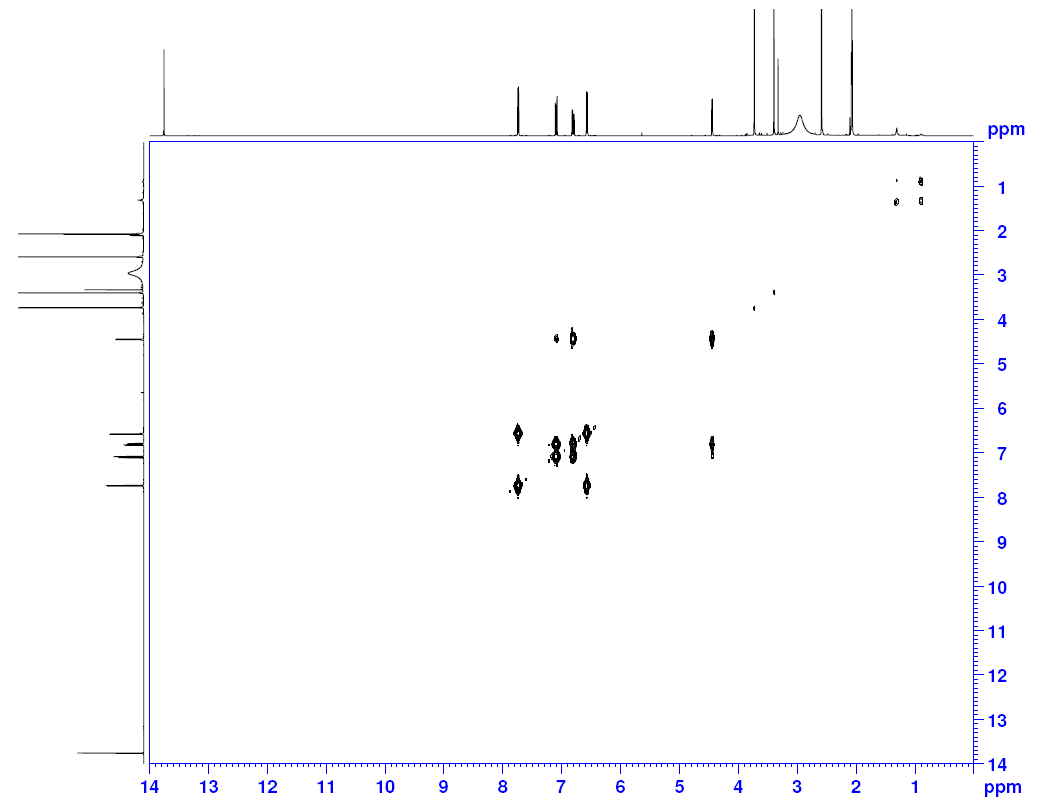


Figure S18. HMBC spectrum of compound **3** in acetone-*d*_6_


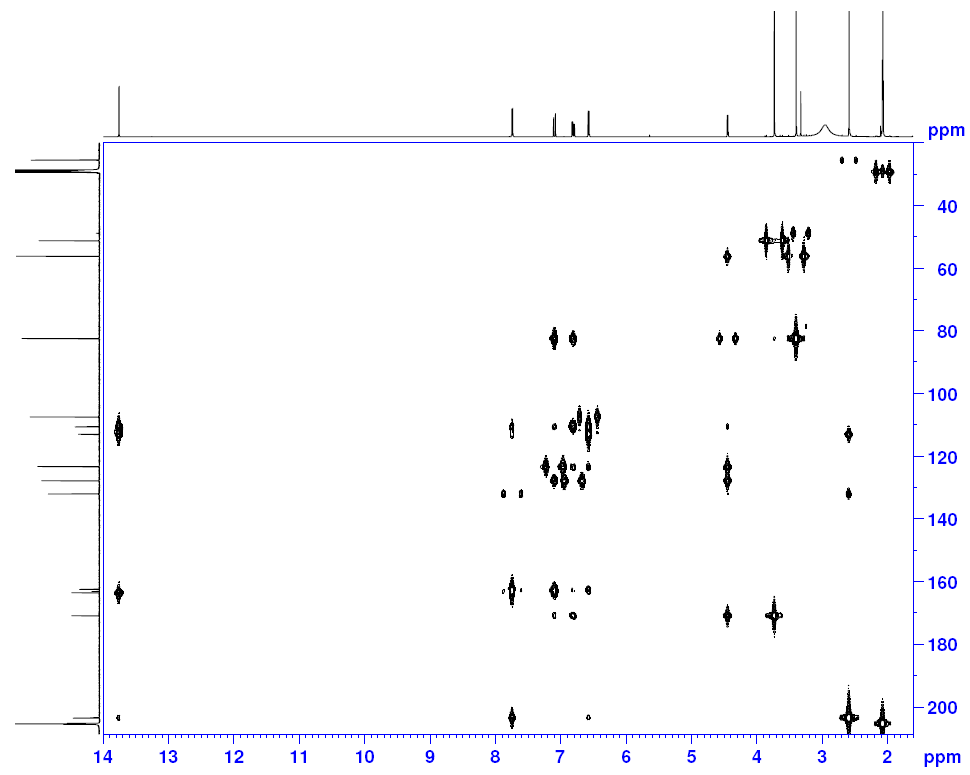

Supplement: Supplementary file 1 [file marinedrugs-16-00045-s001.doc]
